# Treatment of Breakthrough and Refractory Chemotherapy-Induced Nausea and Vomiting

**DOI:** 10.1155/2015/595894

**Published:** 2015-09-03

**Authors:** Rudolph M. Navari

**Affiliations:** ^1^Indiana University School of Medicine, South Bend, IN 46617, USA; ^2^South Bend Medical Services Corporation, 202 Lincoln Way East, Mishawaka, IN 46544, USA

## Abstract

Despite significant progress in the prevention of chemotherapy-induced nausea and vomiting (CINV) with the introduction of new antiemetic agents, 30–50% of patients receiving moderately or highly emetogenic chemotherapy (MEC or HEC) and guideline directed prophylactic antiemetics develop breakthrough CINV. International guidelines recommend the treatment of breakthrough CINV with an agent from a drug class that was not used in the prophylactic antiemetic regimen and recommend using the breakthrough medication continuously rather than using it on an as needed basis. There have been very few studies on the treatment of breakthrough CINV. A recent double-blind, randomized, phase III study suggested that olanzapine may be an effective agent for the treatment of breakthrough CINV. Refractory CINV occurs when patients develop CINV during subsequent cycles of chemotherapy when antiemetic prophylaxis has not been successful in controlling CINV in earlier cycles. Patients who develop refractory CINV should be considered for a change in their prophylactic antiemetic regimen. If significant anxiety exists, a benzodiazepine may be added to the prophylactic regimen. If a refractory patient is receiving HEC, olanzapine may be added to the prophylactic regimen. If the patient is receiving MEC, olanzapine or an NK-1 receptor antagonist may be added to the prophylactic regimen.

## 1. Introduction

Chemotherapy-induced nausea and vomiting (CINV) adversely affects patients' quality of life and may affect patients' treatment decisions [1–3]. The emetogenicity of the chemotherapy administered and specific patient characteristics such as female gender, age, and history of amount of alcohol intake affect patients' risk factors for CINV (Table 1) [3].

Significant and uncontrolled CINV may result in patients returning to the chemotherapy treatment facility one to three days after chemotherapy for rehydration or emesis or nausea control. If CINV cannot be controlled in an outpatient facility, patients may subsequently be treated in an emergency department or require hospitalization [1, 3]. Patients who have an electrolyte imbalance or those who have recently undergone surgery or radiation therapy are at greater risk of experiencing serious complications from CINV [1–3].

The use of 5-hydroxytryptamine-3 (5-HT_3_) receptor antagonists has improved the control of CINV [4, 5]. Additional improvement in the control of CINV has occurred with the use of aprepitant, the first agent available in the drug class of neurokinin-1 (NK-1) receptor antagonists [6], and olanzapine, an antipsychotic agent which blocks multiple neurotransmitters in the central nervous system [7–9].

The primary endpoint used for studies evaluating various agents for the control of CINV has been complete response (CR) (no emesis, no use of rescue medication) over the acute (24 hours after chemotherapy), delayed (24–120 hours), and overall (0–120 hours) periods [3]. The combination of a 5-HT_3_ receptor antagonist, dexamethasone, and a NK-1 receptor antagonist has improved the control of emesis in patients receiving either highly emetogenic chemotherapy (HEC) or moderately emetogenic chemotherapy (MEC) over a 120-hour period following chemotherapy administration [5, 6]. Many of these same studies have measured nausea as a secondary endpoint, but nausea has not been well controlled [10, 11].

The use of effective antiemetic agents in various clinical settings has been described in established guidelines from the Multinational Association of Supportive Care in Cancer (MASCC), the European Society of Medical Oncology (ESMO) [12], the American Society of Clinical Oncology (ASCO) [13], and the National Comprehensive Cancer Network (NCCN) [14].

The purpose of this review is to provide a clinical approach to treatment of breakthrough CINV and refractory CINV based on the available data in the literature as well as the recommendations provided by established guidelines [12–14].

## 2. Types of CINV

CINV can be attributed to several treatment-related factors, including the environment in which chemotherapy is administered, the emetogenicity of the chemotherapy, the dosage of the emetogenic agents, and patient-related factors [15–17]. There are multiple mechanisms involved in the development of CINV, and the mechanisms appear to be different for CINV occurring in the first 24 hours after chemotherapy in contrast to that which occurs in the period of one to five days after chemotherapy. In order to differentiate these mechanisms, five categories are used to classify CINV: acute, delayed, anticipatory, breakthrough, and refractory.

### 2.1. Acute CINV

CINV occurring in the first 24 hours after chemotherapy is considered acute CINV and is primarily due to the activation of the serotonin receptors in the gastrointestinal tract. The combination of the 5-HT_3_ receptor antagonists, dexamethasone, and the NK-1 receptor antagonists has been very effective in the prevention of acute CINV [3–5, 12–14].

### 2.2. Delayed CINV

Delayed emesis and/or nausea and/or vomiting that develop more than 24 hours after chemotherapy administration are known as delayed emesis and/or nausea. For many chemotherapy agents such as cisplatin, doxorubicin, or cyclophosphamide, delayed CINV intensity peaks at two to three days post chemotherapy administration and may persist for five days after chemotherapy [1, 12–15]. Delayed CINV is mediated primarily by substance P receptors and the NK-1 receptor antagonists have been effective in the prevention of delayed CINV [6]. Significant predictive factors include the dose and the emetogenicity of the chemotherapeutic agent, patient's gender and age, and protection against nausea and vomiting in previous cycles of chemotherapy [1, 15].

### 2.3. Anticipatory CINV

Patients may develop a conditioned response known as anticipatory nausea and/or emesis if they experience CINV after chemotherapy administration despite prophylactic antiemetics. Emesis and/or nausea which occurs prior to the administration of chemotherapy in future chemotherapy cycles and is attributed to the adverse memory of prior CINV is considered anticipatory CINV. Incidence rates for this type of nausea and vomiting range from 10 to 45%, with nausea occurring more frequently [1, 15].

### 2.4. Breakthrough CINV

Breakthrough CINV is vomiting and/or nausea that occurs within five days of chemotherapy administration after the use of guideline directed prophylactic antiemetic agents. This type of CINV usually requires immediate treatment or requires “rescue” with additional antiemetics.

### 2.5. Refractory CINV

Refractory CINV is defined as vomiting and/or nausea occurring after chemotherapy in subsequent chemotherapy cycles after guideline directed prophylactic antiemetic agents have failed in earlier cycles [1, 12–14, 17].

## 3. Treatment of Breakthrough CINV

Breakthrough CINV, nausea and emesis which occur despite adequate antiemetic prophylaxis, remains a significant clinical patient problem [16–19] even with the improved control of acute and delayed CINV. There have been very few clinical trials on the treatment of breakthrough CINV [12–14]. When breakthrough CINV occurs, treatment may be considered with agents such as dopamine antagonists or benzodiazepines [12, 13], metoclopramide may be substituted for the 5HT_3_ receptor antagonist in the prophylactic regimen, or a different 5-HT_3_ may be used in the prophylactic regimen according to the MASCC [12] and ASCO [13] guidelines. The NCCN [14] guidelines suggest treating breakthrough CINV with an agent from a drug class that was not used in the prophylactic regimen and recommend continuing the breakthrough medication if nausea and vomiting are controlled.

The treatment of established nausea and vomiting has not been well studied [20, 21], and the agents that have been used for the prevention of CINV have not demonstrated significant efficacy in the treatment of CINV [20, 21]. In 2012, the National Cancer Institute Physician Data Query (PDQ) for supportive care states that there is no known effective therapy for treatment of nausea and vomiting which occurs after chemotherapy [21].

### 3.1. Phase II Studies

The prevention of breakthrough CINV has slowly improved with the introduction of the serotonin receptor antagonists [22–24], the addition of dexamethasone [24, 25], and the addition of NK-1 receptor antagonists [26] to the prophylactic regimens. There have been few studies, however, on the treatment of breakthrough CINV.

Jones et al. [27] reported in a phase II study on 96 patients who received MEC or HEC and guideline directed prophylactic antiemetics. Thirty-nine patients (41%) developed breakthrough nausea and/or vomiting requiring treatment. Twenty-seven of the 39 patients completed a questionnaire on the effectiveness of either oral prochlorperazine (24 patients) or an oral 5-HT_3_ receptor antagonist (3 patients) as a treatment for breakthrough CINV. There was a median nausea reduction of 75% for either treatment after four hours. No data were reported after the four-hour period.

In an additional phase II study, 33 patients who developed breakthrough nausea and/or emesis after receiving MEC or HEC and antiemetic prophylaxis applied a transdermal gel consisting of lorazepam, diphenhydramine, and haloperidol to the palmar surface of their wrists [28]. They were retrospectively interviewed with a questionnaire, and 27 of 33 patients reported a decrease in their nausea and/or vomiting within a four-hour period. Data beyond four hours were not reported.

Chanthawong et al. [29] recently reported a phase II prospective open label clinical trial in which cancer patients diagnosed with solid tumors were enrolled to receive at least one cycle of HEC. Each patient received ondansetron, corticosteroids, and metoclopramide as prophylactic antiemetics. Forty-six patients developed breakthrough CINV and were treated with olanzapine, 5 mg orally every 12 hours for two doses. Patients were evaluated every six hours for 24 hours. The CR of breakthrough emesis, the control of retching, and nausea control were 60.9%, 71.7%, and 50.0%, respectively. Adverse events were mild and tolerable including dizziness, fatigue, and dyspepsia.

### 3.2. Phase III Study

Olanzapine and metoclopramide are two agents that have been recommended by the international guidelines [12–14] for the treatment of breakthrough CINV. These two agents were studied and compared in a double-blind, randomized phase III trial [30]. Chemotherapy naïve patients receiving HEC (cisplatin, ≥70 mg/m^2^, or doxorubicin, ≥50 mg/m^2^, and cyclophosphamide, ≥600 mg/m^2^) and guideline directed prophylactic antiemetics, dexamethasone (12 mg IV), palonosetron (0.25 mg IV), and fosaprepitant (150 mg IV) before chemotherapy and dexamethasone (8 mg p.o. daily, days 2–4) after chemotherapy, who developed breakthrough CINV, were randomized to receive olanzapine, 10 mg orally daily for three days, or metoclopramide, 10 mg orally TID for three days. In July, 2013, in order to reduce neurological toxicities, the European Medicines Agency recommended that the adult dose of metoclopramide be restricted to ≤30 mg/day for five days. Patients were monitored for emesis and nausea for the 72 hours after initiating the olanzapine or the metoclopramide.

Two hundred seventy-six patients (median age 56 yrs, range 38–79; 43 females; ECOG PS 0, 1) consented to the protocol. One hundred twelve patients developed breakthrough CINV and 108 were evaluable. Thirty-nine of 56 (70%) patients receiving olanzapine had no emesis compared to 16 of 52 (31%) patients with no emesis for patients receiving metoclopramide (*P* < 0.01) during the 72-hour observation period. Patients with no nausea (0, 0–10 visual analogue scale) during the 72-hour observation period were: olanzapine: 68% (38 of 56); metoclopramide 23% (12 of 52) (*P* < 0.01). During the 72-hour observation period, there were no observed or reported Grade 3 or 4 toxicities. For patients receiving HEC who were given guideline directed prophylactic antiemetics and subsequently developed breakthrough CINV, the use of olanzapine was significantly better than metoclopramide in the treatment of nausea and emesis over a 72-hour period [30].

### 3.3. Olanzapine

Olanzapine has been reported to be an effective agent in the treatment of acute and chronic nausea [31–34]. Fifteen advanced cancer patients with opioid-induced nausea were treated effectively with olanzapine [31]. A patient's chronic nausea was improved with the use of olanzapine [32], and, in six patients receiving palliative care, Jackson and Tavernier [33] found olanzapine to be effective for intractable nausea due to opioids, neoplasm, and/or medications. Olanzapine was reported to be very effective in controlling refractory nausea and vomiting in two patients with advanced cancer [34].

The mechanism of action of olanzapine involves the blocking multiple neurotransmitter receptors including dopaminergic at D_1_, D_2_, D_3_, and D_4_ brain receptors, serotonergic at 5-HT_2a_, 5-HT_2c_, 5-HT_3_, and 5-HT_6_ receptors, catecholamines at alpha_1_ adrenergic receptors, acetylcholine at muscarinic receptors, and histamine at H_1_ receptors [35, 36]. Olanzapine has five times the affinity for 5-HT_2_ receptors compared to D_2_ receptors [35, 36]. Dopamine and serotonin are known mediators of CINV [35, 36]. Olanzapine appears to have activity in controlling both acute and delayed emesis and nausea and may exert much of its antiemetic effect in the central nervous system at multiple cortical receptors. It is not known whether a peripheral effect may also exist. Olanzapine blocks the serotonin mediated 5-HT_2c_ receptor, a receptor which has been shown to mediate antiemetic activity in animal models (ferret cisplatin-induced emesis and cisplatin-induced anorexia in the hypothalamus of rats) [37, 38]. The effect of olanzapine on this receptor as well as other dopamine and serotonin receptors may explain its efficacy.

In a number of phase II and phase III studies [8, 9, 39–41], olanzapine has also been shown to be a safe and effective agent for the prevention of CINV. A phase II trial involving 30 patients receiving MEC and HEC demonstrated that a combination of olanzapine, granisetron, and dexamethasone was effective in controlling emesis and nausea [39]. In forty patients receiving MEC or HEC, the combination of olanzapine, palonosetron, and dexamethasone was effective in the prevention of CINV [40].

Tan et al. [8] studied the use of olanzapine as a prophylactic agent in patients receiving either MEC or HEC by adding olanzapine to a prophylactic regimen of azasetron and dexamethasone. In a total patient group of 229 patients receiving either MEC or HEC, CR was significantly improved in the patients receiving olanzapine, azasetron, and dexamethasone compared to patients receiving azasetron and dexamethasone in the delayed and overall periods. Olanzapine improved the CR of delayed and overall CINV and quality of life in patients receiving MEC and HEC [8].

International guidelines have recommended the use of a 5-HT_3_ receptor antagonist, dexamethasone, and an NK-1 receptor antagonist as the prophylactic antiemetic regimen for patients receiving HEC [12–14]. In order to further investigate the effectiveness of olanzapine as a prophylactic antiemetic for patients receiving HEC, a phase III study was performed to compare the effectiveness of olanzapine versus aprepitant for the prevention of CINV in patients receiving HEC [9].

Chemotherapy naïve patients receiving HEC (cisplatin or an anthracycline plus cyclophosphamide) were randomized to a prophylactic antiemetic regimen of olanzapine, palonosetron, and dexamethasone or to a prophylactic regimen of aprepitant, palonosetron, and dexamethasone. For the 121 patients receiving the olanzapine regimen and the 120 patients receiving the aprepitant regimen, there was no significant difference in CR in the acute, delayed, or overall period. The number of patients with no nausea (0, scale 0–10, M.D. Anderson Symptom Inventory) was significantly higher in the delayed and overall periods for the patients who received the olanzapine regimen. Olanzapine appeared to be an effective agent for the control of nausea in patients receiving HEC [9].

In a randomized, double-blind, placebo-controlled trial, 44 patients scheduled to receive MEC or HEC received a 5-HT_3_ receptor antagonist, dexamethasone, and a NK-1 receptor antagonist. Patients were then randomized to receive 5 mg of olanzapine daily for 6 days beginning on the day before chemotherapy or placebo. CR and no nausea were significantly improved in the patients receiving olanzapine [41].

Recent reviews [7, 42, 43] have concluded that antiemetic regimens including olanzapine are more effective in reducing CINV compared to regimens that do not include olanzapine.

### 3.4. Guideline Recommendations

Current guidelines suggest the use of a phenothiazine, metoclopramide, dexamethasone, butyrophenones, cannabinoids, anticholinergics, or olanzapine for the treatment of breakthrough nausea and vomiting [14]. A 5-HT_3_ receptor antagonist may also be effective unless a patient presents with nausea and vomiting which was developed following the use of a 5-HT_3_ receptor antagonist as prophylaxis for chemotherapy- or radiotherapy-induced emesis. It is very unlikely that breakthrough nausea and vomiting will respond to an agent in the same drug class after unsuccessful prophylaxis with an agent with the same mechanism of action. Agents which are successful in treating a patient's breakthrough CINV should be given routinely for a period of time rather than on an as needed basis.

The NCCN guidelines [14] state that patients who develop nausea or vomiting after chemotherapy (days 1 to 5) despite adequate prophylaxis should be considered for treatment with a three-day regimen of oral olanzapine based on the results of a phase III clinical trial [30]. This recommendation was supported by a recent review by Bradford and Glode [44].

It is important to note that aprepitant has been approved as an additive agent to a 5-HT_3_ receptor antagonist and dexamethasone for the prevention of CINV. It has not been studied and should not be used to treat breakthrough nausea and vomiting.

## 4. Clinical Approach to Refractory CINV

Patients who develop CINV during subsequent cycles of chemotherapy when antiemetic prophylaxis has not been successful in controlling CINV in earlier cycles should be considered for a change in the prophylactic antiemetic regimen. If anxiety is considered to be a major patient factor in the CINV, a benzodiazepine such as lorazepam or alprazolam can be added to the prophylactic regimen. If the patient is receiving HEC, olanzapine (days 1 to 4) can be substituted for aprepitant or fosaprepitant in the prophylactic antiemetic regimen [9]. If the patient is receiving MEC, aprepitant or fosaprepitant can be added to the palonosetron and dexamethasone antiemetic regimen [45].

Vig et al. [46] reported a retrospective study on the efficacy of the addition of olanzapine in adults experiencing refractory CINV. Thirty-three adults who experienced CINV refractory to guideline directed prophylactic antiemetics and experienced breakthrough treatment with dopamine antagonists and benzodiazepines received olanzapine, 5–10 mg as an addition to the prophylactic antiemetics in a subsequent chemotherapy cycle. Failure was defined as >5 emesis events in 24 hours or more than 10 cumulative doses of rescue antiemetics following the first olanzapine dose per treatment cycle. The addition of olanzapine demonstrated an overall success rate of 70%. The success rate appeared to be independent of the chemotherapy emetogenicity (HEC versus MEC), age, or prophylaxis with a serotonin antagonist, plus a corticosteroid, and aprepitant or a serotonin antagonist alone.

NCCN guidelines [14] have recommended the use of olanzapine, palonosetron, and dexamethasone [42] as an alternative first-line prophylactic antiemetic regimen for patients receiving HEC. This regimen may be useful in patients who experience refractory CINV and need a revision in their prophylactic antiemetic regimen [42, 46].

## 5. Conclusions

Breakthrough CINV can occur in 30–40% of patients receiving MEC or HEC despite the use of guideline directed prophylactic antiemetics. Guidelines [12–14] have recommended treatment with phenothiazines, metoclopramide, butyrophenones, corticosteroids, cannabinoids, anticholinergics, 5-HT_3_ receptor antagonists, and olanzapine. Agents which have not been part of the prophylactic antiemetic regimen should be employed and given continuously rather than on an as needed basis. A recent randomized, double-blind, phase III clinical trial demonstrated that olanzapine was very effective in the treatment of breakthrough CINV in patients receiving HEC and guideline directed prophylactic antiemetics.

Patients who experience refractory CINV should have a change in their prophylactic antiemetic regimen prior to the next chemotherapy cycle. Olanzapine appears to be an effective agent either as an additive to the previous prophylactic antiemetic regimen or in combination with palonosetron and dexamethasone. An antianxiety agent may be added to the prophylactic regimen if anxiety appears to be a major component of the patient's CINV. In patients with refractory CINV receiving MEC, olanzapine or a NK-1 receptor antagonist may be added to the prophylactic antiemetic regimen.

## Figures and Tables

**Table 1:** Patient-related risk factors for emesis following chemotherapy.

Major Factors	Minor factors
Female	History of motion sickness
Age <50 years	Emesis during past pregnancy
History of low prior chronic alcohol intake (<1 ounce of alcohol/day)	Anxiety
History of previous chemotherapy-induced emesis	
